# Prospective observational study to evaluate the clinical safety of the fixed-dose artemisinin-based combination Eurartesim® (dihydroartemisinin/piperaquine), in public health facilities in Burkina Faso, Mozambique, Ghana, and Tanzania

**DOI:** 10.1186/s12936-015-0664-9

**Published:** 2015-04-15

**Authors:** Rita Baiden, Abraham Oduro, Tinto Halidou, Margaret Gyapong, Ali Sie, Eusebio Macete, Salim Abdulla, Seth Owusu-Agyei, Abdunoor Mulokozi, Alex Adjei, Esperanca Sevene, Guillaume Compaoré, Innocent Valea, Isaac Osei, Abena Yawson, Martin Adjuik, Raymond Akparibo, Bernhards Ogutu, Gabriel Leonard Upunda, Peter Smith, Fred Binka

**Affiliations:** INDEPTH Network, Accra, Ghana; Navrongo Health Research Centre, Navrongo, Ghana; Nanoro Health Research Centre, Nanoro, Burkina Faso; Dodowa Health Research Centre, Dodowa, Ghana; Nouna Health Research Centre, Nouna, Burkina Faso; Centro de InvestigaçãoemSaúde de Manhiça, CISM, Manhiça, Mozambique; Ifakara Health Institute, Ifakara, Tanzania; Kintampo Health Research Centre, Kintampo, Ghana; Ministry of Health, Dar es Salaam, Tanzania; London School of Hygiene & Tropical Medicine, London, UK; University for Health and Allied Sciences, Ho, Ghana

**Keywords:** Cohort event monitoring, Eurartesim®, Safety monitoring, Electrocardiogram, QTc prolongation

## Abstract

**Background:**

The World Health Organization recommends artemisinin-based combination (ACT) for the treatment of uncomplicated malaria. Post-licensure safety data on newly registered ACT is critical for evaluating their risk/benefit profile in malaria endemic countries. The clinical safety of the newly registered combination, Eurartesim®, following its introduction into the public health system in four African countries was assessed.

**Methods:**

This was a prospective, observational, open-label, non-comparative, longitudinal, multi-centre study using cohort event monitoring. Patients with confirmed malaria had their first dose observed and instructed on how to take the second and the third doses at home. Patients were contacted on day 5 ± 2 to assess adherence and adverse events (AEs). Spontaneous reporting of AEs was continued till day 28. A nested cohort who completed full treatment course had repeated electrocardiogram (ECG) measurements to assess effect on QTc interval.

**Results:**

A total of 10,925 uncomplicated malaria patients were treated with Eurartesim®. Most patients,95% (10,359/10,925), did not report any adverse event following at least one dose of Eurartesim®. A total of 797 adverse events were reported. The most frequently reported, by system organ classification, were infections and infestations (3. 24%) and gastrointestinal disorders (1. 37%). In the nested cohort, no patient had QTcF > 500 ms prior to day 3 pre-dose 3. Three patients had QTcF > 500 ms (509 ms, 501 ms, 538 ms) three to four hours after intake of the last dose. All the QTcF values in the three patients had returned to <500 ms at the next scheduled ECG on day 7 (470 ms, 442 ms, 411 ms). On day 3 pre- and post-dose 3, 70 and 89 patients, respectively, had a QTcF increase of ≥ 60 ms compared to their baseline, but returned to nearly baseline values on day 7.

**Conclusion:**

Eurartesim® single course treatment for uncomplicated falciparum malaria is well-tolerated. QT interval prolongation above 500 ms may occur at a rate of three per 1,002 patients after the third dose with no association of any clinical symptoms. QT interval prolongation above 60 ms was detected in less than 10% of the patients without any clinical abnormalities.

**Electronic supplementary material:**

The online version of this article (doi:10.1186/s12936-015-0664-9) contains supplementary material, which is available to authorized users.

## Background

The World Health Organization (WHO) recommends artemisinin-based combination therapy (ACT) for the treatment of patients with uncomplicated malaria [1]. In controlled trials in Africa, ACT has been found to be generally safe [2-10]. In October 2011, the European Medicines Agency (EMA) approved a new ACT, Eurartesim®, for the treatment of uncomplicated malaria caused by *Plasmodium falciparum* [11]. Eurartesim® is a fixed-dose combination of dihydroartemisinin (DHA) and piperaquine phosphate (PQP) in film-coated tablets. DHA is a fast acting anti-malarial and PQP is an orally active bisquinoline, structurally related to chloroquine, highly lipophilic with a half-life of about 22 days [12,13].

A high treatment success (>95%) with Eurartesim® was demonstrated in pivotal trials conducted in Africa and Asia, but in some studies electrocardiogram (ECG) monitoring showed a prolonged QTc interval in some patients. Some other anti-malarials have also been shown to produce a transient prolongation of QT interval during treatment, without significant clinical relevance to the patient [14-16]. At the time of registration of Eurartesim®, the Committee for Medicinal Products for Human Use (CHMP) of EMA considered that the safety database for the drug was insufficient to determine the frequency of QT prolongation and the risk of arrhythmias after taking Eurartesim®, though non-clinical data showed the torsado-genic cardiotoxicity of Eurartesim®to be lower than that of chloroquine and similar to that of mefloquine and artemether + lumifantrine [17,18]. As part of the post-registration requirements, EMA requested a risk management plan, including safety data from a phase IV safety study. Such post-licensure data were considered important for evaluating the risk/benefit profile of the drug to inform decisions on the use of the drug by policy makers, globally and at national levels. The requested Phase IV study has been conducted by the INDEPTH Effectiveness and Safety platform (INESS) [19].

### The INDEPTH effectiveness and safety study (INESS) platform

The INESS platform was set up in 2009 to facilitate the conduct of large phase IV studies of newly registered anti-malarials in Africa, to provide timely country-specific safety and effectiveness data to assist in the formulation of national and global policies. The platform comprises eight Health and Demographic Surveillance Sites (HDSS) of the INDEPTH Network in four African countries (Rufiji and Ifakara in Tanzania, Nouna and Nanoro in Burkina Faso, Navrongo, Kintampo and Dodowa in Ghana and Manhica in Mozambique). All four countries are official members of the WHO pharmacovigilance programme, with established spontaneous reporting systems, although the reporting rates are very low. An independent scientific review committee oversees the scientific activity of INESS. Prior to the introduction of Eurartesim, data collection for patients prescribed available anti-malarials had been ongoing on the platform, using structured questionnaires adapted from the national spontaneous reporting form for the collection of adverse events (AE) after drug treatment, with support from the WHO Collaborating Centre for Advocacy and Training in Pharmacovigilance. This study presents the initial findings of the safety of Eurartesim® following its introduction into the four African countries.

## Methods

### Study setting

The study protocol was approved by an Independent Scientific Review Panel composed by experts in pharmacovigilance, tropical medicine and statistics. The study was conducted in seven HDSS (Rufiji, Nouna, Nanoro, Navrongo, Kintampo, Dodowa and Manhica), with an estimated total population under demographic surveillance of about 750,000. Malaria is endemic in all the sites*,* with *P falciparum* accounting for more than 90% of malaria infections. At the time of the study, the first-line drugs for uncomplicated malaria in these countries were artesunate + amodiaquine and artemether + lumefantrine, and in Ghana, also DHA + PQP. In Tanzania, DHA + PQP is recommended as the first-line alternate [20-26].

### Study design

The study was designed as a cohort event monitoring (CEM) study - a prospective, observational, open-label, non-comparative, multi-centre study. All patients visiting the 41 selected health facilities were screened by a health worker for malaria symptoms (presence or history of fever within two days, chills, headache, general malaise and loss of appetite), and malaria was diagnosed using an approved malaria rapid diagnostic test (RDT), as per the national guidelines and recommended by WHO. An eligible patient (or their guardian) was invited to participate in the study after uncomplicated malaria was diagnosed and signed informed consent or assent (for children between age 12 and 18 years) was obtained. Eligible patients were aged over six months, weighed more than 5 kg and were able to take oral medications. Patients were excluded if they had any of the following: intake of DHA/PQP in the previous four weeks; known allergy to artemisinin or piperaquine; known to be pregnant or lactating; severe or complicated malaria; taking medicinal products that prolong the QTc interval, including antiarrhythmics and neuroleptics; family or personal history of cardiac arrhythmia/QT prolongation (including congenital long QT syndrome, arrhythmia), and family history of sudden unexplained death. Also patients were excluded from the ECG sub-study (see below) if they had QTc interval greater than 450 milliseconds, with either Bazett or Fridericia correction, in the initial ECG.

### Enrolment and study procedure

Each eligible participant had a detailed clinical examination and any significant medical history was recorded. A microscopic slide for malaria parasites was prepared, time of intake of the last meal was recorded and case report forms (CRF) were corrected. For eligible patients, the first dose of Eurartesim® was administered with water under supervision by a study staff member. The patient was observed for about an hour for any AE. During this time, the patient was encouraged not to eat any fatty or high calorie foods. Patients were instructed to take the remaining two doses at home with water on days 2 and 3, about three hours before or after meals. Patients were taught how to use diary cards and instructed how to document time and type of food before and after intake of the remaining two doses. Each patient was included once in the study. Patients who vomited within 30 minutes of administration of the first dose were re-treated with the same dosage, and if vomiting occurred within 30 to 60 minutes, half a dose was re-administered. Re-dosing was not attempted more than once. Dosing was in four weight bands as shown in Table 1. Pregnancies discovered within 28 days of Eurartesim® intake were followed at least every three months and after delivery up to 14 weeks to assess pregnancy outcome.

**Table 1:** **Doses of Eurartesim® administered according to patient weight**

**Body weight (kg)**	**Daily dose (mg)**	**Number of tablets per dose**	
		**20/160 mg DHA/PQ**	**40/320 mg DHA/PQ**
**5-12**	20/160 mg DHA/PQP	1	
**13-23**	40/320 mg DHA/PQP		1
**25-35**	40/320 mg DHA/PQP		2
**36-74**	40/320 mg DHA/PQP		3
**>74**	40/320 mg DHA/PQP		4

Patients who, in addition, consented to be part of a nested cohort was selected if QTcF interval was less than 450 ms and had an intensive laboratory assessment, samples for pharmacokinetic analysis. A series of ECGs monitoring at the baseline (day 1 pre-dose 1), day 3 pre-dose 3, day 3 post-dose 3 and on day 7 at the health facility. The first dose of Eurartesim® was given at the clinic also under “Direct Observed Treatment” three hours before or after meals and observed for an hour. Dose 2 was taken at home and dose 3 at the health facility.

### Follow-up for any adverse events

All patients had a scheduled follow up on day 5 (±2 days) either by telephone or by a visit, by field supervisors or health agents trained by the research team to solicit AE. Record was made of whether any symptoms of malaria remained and of any AE that had occurred since Eurartesim® was administered. Patients were contacted again on day 28 to ascertain any further AE. Patients who had any medical event of concern, or whose symptoms worsened between intake of the first dose and within 28 days of intake of Eurartesim®, were asked to visit the nearest health facility or to immediately contact the site investigators, using the telephone numbers on the consent form. If any patient reported a cardiac event, an ECG was performed and the trace was inspected for QTc prolongation or other abnormalities. Any patient reporting an adverse event deemed to be serious was given recommended care in line with the national standard in that country. Each site had a local pharmacovigilance monitoring committee, which regularly reviewed the listings of AE collected during the study. They also received notification of serious adverse events (SAE), adverse events of special interest (cardiac toxicity, neurological toxicity and photo-toxicity) and all AE classified as severe. These committees were charged with identifying possible safety signals and communicating any such signals immediately to the study investigators. National regulatory authorities and ethics committees were also notified of any AE classified as serious.

### Nested cohort

#### ECG recording and interpretation

Patients in the nested cohort had more detailed investigations, primarily to assess any effect of Eurartesim® on the QTc interval duration. Blood samples were also taken for pharmacokinetic studies. After clinical examination, all patients in the nested cohort had baseline ECGs, in triplicate, using 12-leads digitalized ELI 150 Cardiograph®. Tracings were done at least three hours before or after food intake and the three recordings were taken in a quiet room, with an interval between the readings of 1 to 2 minutes. ECGs were read by trained and ECG-certified study clinicians (by Cardiabase) before the first dose of Eurartesim® was administered. The QTcF was automatically calculated and manually verified by the study clinician. Participants with an average QTcF of ≥ 450 ms were excluded from the study and prescribed alternative anti-malarial medicines, as per the national guidelines. Electronic copies of all ECGs were sent to the Cardiabase laboratory in France [27] for interpretation by a certified cardiologist. Study clinicians notified Cardiabase regarding any patients for whom ECG reports were required within 24 hours for purposes of clinical management. A further ECG was done immediately prior to dose 3 of Eurartesim® and pharmacokinetic (PKa) blood samples were taken before the day 3 dose was administered. The patients were observed for three to four hours after dose 3 and then post-dose three ECGs (triplicate) were performed, followed by collection of PKa blood samples. Participants whose pre-dose 3 QTc interval was ≥ 500 ms were observed for six hours and ECGs were repeated until the QTc interval was less than 480 ms before the third dose was administered. Alternative anti-malarial medicines were administered if the QTc interval persistently remained above 480 ms. On day 7, ECGs were repeated for each participant.

Reading of the set of ECGs for a given patient was performed by the same cardiologist. The reader was blinded with respect to the timing and the day of the ECG recording. A computer-assisted, semi-automatic, on-screen measurement of the digital ECG waveform was used for the reading (ECG Manager©). The QT interval measurement was edited from the global superimposed median beats. Each median beat was mathematically derived from the available recording of the corresponding lead. The 12 individual median beats were graphically displayed as temporally aligned and overlapped one to another. Global interval measurement was subsequently defined as the interval from the earliest onset observed on any of the 12 superimposed lead to the latest offset observed on any of the 12 leads, in accordance with the American College of Cardiology/American Heart Association recommendations. Quality control checks of the readers were conducted during the study, including the assessment of the intra-reader and inter-reader variability, to assure the quality of the results. Copies of all ECG confirmed reports were sent back to the study clinicians and stored in the central database.

### Outcome

The main outcome of interest was clinical safety evaluated through the analysis of AE after administration of Eurartesim® treatment, including AEvidentified in the referring health facilities or spontaneously reported by the patients as occurring within 28 days after the first medication . In the nested cohort, cardiotoxicity from ECG findings (QT corrected by Fridericia”s formula) was the main safety outcome. AE of special interest (AESI) – relating to cardiotoxicity - were also an outcome of interest in both groups.

### Sample size

It was planned to collect data on Eurartesim® treatment of approximately 10,000 cases of uncomplicated malaria. This number of cases allowed identification of at least one AE occurring at a frequency of one in 3000 with a 95% probability. In the nested cohort, a sample of 1,000 gave a probability of 0. 865 of observing at least one cardiac event of interest (i. e. palpitations, fainting/syncope, pounding/pain in the chest area, seizures) that might be related to QT interval prolongation, assuming the true rate was at least 3/1,000.

### Data entry and statistical analysis

Data were double-entered and verified using OpenClinica software. Statistical analyses were performed using the software package STATA® (version 11. 2). Descriptive analyses were conducted of all data recorded at study entry. AEs were documented as described by the participant or caregiver, reviewed and coded using version 13. 1 of the Medical Dictionary for Regulatory Activities (MedDRA®). All events reported were grouped by MedDRA® System Organ Class (SOC) classification. The estimates of the incidence of AEs were based on crude rates, with no attempt to carry out causality assessment of individual cases. In the nested cohort, the QTc interval was evaluated after correcting for the heart rate with Fridericia’s formula (QTcF = QT/RR^1/3^). Descriptive analysis of the mean QTcF was done and the mean difference between the baseline and day 3 pre-dose, day 3 post-dose and day 7 was computed. The primary outcome of interest was QTcF > 500 ms after intake of the first dose of Eurartesim® or a change of QTcF(ΔQTcF) ≥ 60 ms from baseline at each scheduled ECG. Patients who had increased QTcF (≥500 ms), or a prolonged interval of more than 60 ms at any point of their scheduled ECG, were followed through today 7 for QTc outcome [28,29]. Blood biochemistry and pharmacokinetic sample analysis will be presented elsewhere.

### Ethics

Written informed consent was obtained from all patients before performance of any study-related activity. The protocol was approved by the national ethics committees in Ghana, Burkina Faso, Tanzania and Mozambique and institutional review boards at each participating site. The study was registered with Clinicaltrials. gov (NCT02199951) prior to enrolment of the first subject.

## Results

### Cohort composition

**Figure 1:**
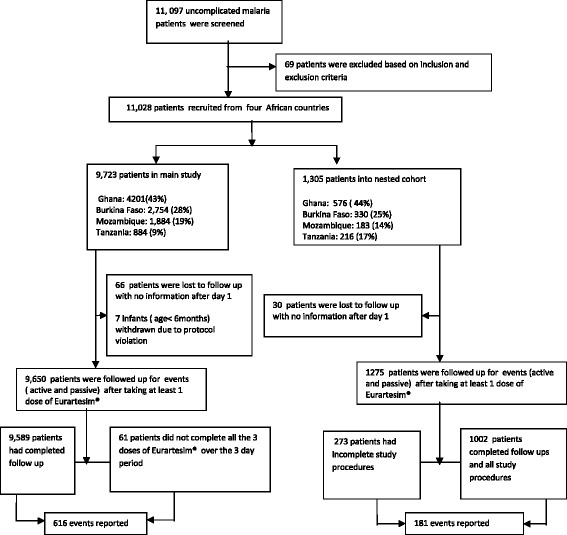
Patient flow.

11,097 patients with uncomplicated malaria were screened over a period of 10 months, (September 2013 to June 2014) and 11,028 were enrolled. 9,723 (88%) were recruited into the main study and 1,305 (12%) into the nested cohort (Figure 1). 96 patients (0. 9%) were lost to follow-up with no information after intake of the first dose at the health facility and seven children were withdrawn, being under six months old. Sixty-one patients in the main study did not complete the full course of Eurartesim® over the three day period due to vomiting after re-dosing on day 1 (43) or worsening of their presenting symptoms (18).

In the nested cohort, 273 patients were not included in the analysis. Eleven had baseline mean QTcF > 450 ms, 262did not have either the required number of eight ECGs (triplicate on day 1, single on day 3 pre-dose 3, triplicateon day 3 post-dose 3 and a single ECG on day 7) or the required four PKa samples.

### Participants with complete treatments and study procedures

10,591 (1,002 in the nested cohort) completed a full course of Eurartesim® over a three-day period and had complete follow up (Figure 1). The largest number of patients was recruited in Ghana, followed by Burkina Faso, Mozambique and Tanzania (Table 2). About half of the patients were male and about half were under the age of six years. *Plasmodium falciparum* infection was diagnosed by RDT in all except 30 patientswho were diagnosed according to WHO recommendations on presumptive diagnosisof malaria. 7,391 had microscopic slide reading, and, of these, 44% had parasite densities in excess of 5,000/μL. Sixty-four percent (6,807) of patients received concomitant medications mainly analgesics (56%) and haematinics (15%) (Additional file 1). Sixty-six percent were followed up on day 5 ± 2 person and the rest by telephone. On day 28, 61% were followed up at home and the remainder by phone contact.

**Table 2:** **Demographic and baseline clinical characteristics of participants who took all the three doses of Eurartesim® and completed study procedures**

	**Main study**	**Nested cohort**	**Total n (%)**
**Variable**	**N = 9,589**	**1,002**	**10,591**
**Country (n (%))**			
Burkina Faso	2,718 (28. 3)	299 (29. 8)	3,017 (28. 5)
Ghana	4,119 (43. 0)	444 (44. 3)	4,563 (43. 1)
Mozambique	1,876 (19. 6)	89 (8. 9)	1,965 (18. 6)
Tanzania	876 (9. 1)	170 (17. 0)	1,046 (9. 9)
**Gender (n (%))**			
Female	5,068 (52. 8)	519 (51. 8)	5,587 (52. 7)
Male	4,521 (47. 2)	483 (48. 2)	5,004 (47. 3)
**Age group (n (%))**			
<6	4,654 (48. 5)	331 (33. 0)	4,985 (47. 1)
6 - <13	2,612 (27. 2)	398 (37. 7)	3,010 (28. 4)
13- <18	753 (7. 8)	112 (11. 2)	865 (8. 2)
>18	1,570 (16. 4)	161 (16. 1)	1,731 (16. 3)
***Plasmodium*** **positivity (n (%))**			
RDT only	3,170 (33. 0)	-	3,170(29. 9)
Microscopy only	1,405 (14. 6)	274 (27. 4)	1,679 (15. 9)
Both RDT and microscopy	4,984 (52. 0)	728 (72. 6)	5,712(53. 9)
Presumptive diagnosis	30 (0. 3)	-	30 (0. 3)
*Parasite density (/μL) ( P. falciparum) * **(n (%))**			
No parasite observed	37 (0. 6)	8 (0. 8)	45 (0. 6)
<50	421 (6. 6)	80 (8. 0)	501 (6. 8)
50- < 500	1,282 (20. 1)	214 (21. 5)	1,496 (20. 3)
500- < 5,000	1,800 (28. 3)	265 (26. 6)	2,065 (28. 0)
5000- < 50,000	1,477 (23. 2)	268 (26. 9)	1,745 (23. 7)
≥50,000	1,354 (21. 2)	160 (16. 1)	1,514 (20. 6)

*25 patients had other species of *Plasmodium* that was not *falciparum*.

### AEs and AESIs reported

Most patients, 95% (10,359/10,925), who received at least one dose of Eurartesim® did not report any adverse event. 566 patients reported a total of 797AEs, of which 32 were classified as serious (SAEs). The most frequently reported events, classified by SOC allocation, were infections and infestations (3. 24%), gastrointestinal disorders (1. 37%), general disorders and administrative site conditions (0. 76%) and respiratory, thoracic and mediastinal disorders (0. 5%) (Table 3). MedRA classification by preferred term is as shown in Additional file 2. AESI mainly reported under cardiac disorders, were four instances of palpitations and a newly diagnosed case of hypertension (0. 05%) and 26 instances (0. 24%) of skin and subcutaneous tissue disorders, with half presenting with pruritus. Fifty (0. 46%, including 31 episodes of headache, were reported under nervous system disorders. AEs by study site ranged from 46 to 134 per 1000 with Rufiji reporting the highest rate (Additional file 3).

**Table 3:** **Incidence rate of events reported by system organ classification (grouped by MedDRA® coding) in the total cohort (N = 10,925)**

**MedDRA® System organ classification**	**Number of events, (n)**	**Incidence rate per 1,000 (n/N)**
Blood and lymphatic system disorders	15	1. 4
Cardiac disorders	5	0. 5
Congenital, familial and genetic disorders	3	0. 3
Ear and labyrinth disorders	2	0. 2
Eye disorders	8	0. 7
Gastrointestinal disorders	150	13. 7
General disorders and administrative site conditions	83	7. 6
Immune system disorders	1	1. 0
Infections and infestations	354	32. 4
Injury, poisoning and procedural complications	5	0. 5
Metabolism and nutrition disorders	14	1. 3
Musculoskeletal and connective tissue disorders	12	1. 1
Nervous system disorders	50	4. 6
Pregnancy, puerperium and perinal conditions	1	0. 1
Psychiatric disorders	4	0. 4
Renal and urinary disorders	2	0. 2
Reproductive system and breast disorders	2	0. 2
Respiratory, thoracic and mediastinal disorders	59	5. 4
Skin and subcutaneous tissue disorders	26	2. 4
Other	1	0. 1

Of the 32 SAEs reported, 18 (56%) were in participants aged < six years. Six deaths occurred during the study (two each from Ghana and Tanzania, one each from Burkina Faso and Mozambique) (Additional file 4). Details of these deaths are given below:

- A 17-month old boy who was treated with Eurartesim® presented after two weeks with severe dehydration, secondary to acute gastroenteritis. He died within 24 hours of admission. No autopsy was undertaken. The death was assessed as unlikely to be related to intake of Eurartesim®.
- A 5-yearold male who died at home on the 3rd day after treatment. Verbal autopsy suggested probable hypoglycaemia. Severe malaria could not be ruled out. The death was assessed as unrelated to intake of Eurartesim.
- A22-year old female who had intractable vomiting immediately the first dose of Eurartesim® was administered. She was managed as a case of uncomplicated malaria and acute hepatitis B infection with complication of liver failure. No autopsy was performed. The death was assessed as unrelated to intake of Eurartesim.
- A4-year old female who presented as a case of uncomplicated malaria and was treated with Eurartesim® on an out-patient basis. Her condition deteriorated at home and she presented again within 24 hours of recruitment at the health facility with severe anaemia. She died on the way to a higher referral facility for blood transfusion. No autopsy was performed and the death was assessed as unlikely to be related to intake of Eurartesim®.
- A26-year old female, known to be HIV-infected who died of HIV vasculitis on the 3rd day of treatment with Eurartesim®. The death was assessed as unrelated to intake of Eurartesim®.
- A3-year old female who died at home on the 3rd day after recruitment. Verbal autopsy suggested severe malaria. The death was assessed as unlikely to be related to intake of Eurartesim®.

### Nested cohort

A total of 1,002 patients completed three doses of Eurartesim® and had complete cardiac monitoring. The mean QTcF at baseline was 393. 7 ms. On day 3 pre-dose 3, the mean QTcF (411. 2 ms) increased by 18 ms (95% CI 16,19) from the baseline and on day 3 post-dose 3 (415. 8 ms) by 23 ms (95% CI 21,24) from the baseline. On day 7, the mean QTcF (398. 6 ms) had returned to near the baseline value, with a mean difference of 5. 4 ms, (95% CI 4, 7) (Table 4). The findings were similar for QTcB (QT corrected by Bazett’s formula).

**Table 4:** **ECG summary parameters (means in ms) for day 1, day 3 pre-dose, day 3 post-dose and day 7 for treatment with three doses of Eurartesim® [N = 1,002] based on central laboratory readings**

	**PR**	**HR**	**QT**	**QTcB**	**QTcF**	95% *Confidence interval	*P-value
**Visit**							
**Day 1 (baseline)**	134. 2	104. 6	331. 9	429. 6	393. 3	-	-
**Day 3 pre-dose 3**	138. 3	92. 5	355. 4	434. 2	411. 2	-	-
**Day 3 post-dose 3**	140. 5	91. 6	366. 1	444. 1	415. 8	-	-
**Day 7**	137. 6	94. 2	346. 2	428. 2	398. 6	-	-
**Δ Days 1 to 3 pre-dose**	4. 1	−12. 1	23. 5	4. 6	17. 9	16 ,19	<0. 001
**Δ Days 1 to 3 post-dose**	6. 3	−13. 1	34. 2	14. 6	22. 5	21,24	<0. 001
**Δ Days 1 to 7**	3. 3	−10. 3	14. 2	-. 31	5. 3	4,7	<0. 001

*Confidence intervals and *P-values were calculated for the mean change in QTcF.

### QTcFof greater than 500 ms following treatment

No patient had QTcF greater than 500 ms prior to treatment or on day 3 pre-dose 3. On day 3 post-dose (three to four hours after administration of Eurartesim®) three male patients, aged 8, 19 and 24 years had a mean QTcF of more than 500mswith the following values; 509 ms, 501 ms, 538 ms respectively. All QTcF values in the three patients were less than 500 ms on day 7 (470 ms, 442 ms, and 411 ms, respectively). Since no other ECGs were conducted between days 3 and 7, the time during which the QTcF value was above 500 ms is not known.

Two female patients, aged 62 and 41 years, whose QTcF were less than 500msat baseline, on day 3 pre and post dose 3, had a QTcF > 500 ms (501 ms and 532 ms) on day 7. No clinical symptoms or signs were reported during the follow up of these patients and no other ECGs were performed.

### Increase in QTcFof more than 60 ms from baseline on day 3 pre-dose 3

On day 3 pre-dose three, 70 patients had an increase in the QTcF interval of more than 60 ms compared to their baseline measurement. Sixty-five of these (93%) were children under 12 years old. The oldest person was 27 years. The range of prolongation was 60 to 144 ms. On day 7, in all 70 patients, the mean QTcF interval was less than 60 ms above their baseline measurements – the average increase was 37. 7 ms in males and 27. 9 ms in females.

On day 3 post-dose 3, 89 patients had a QTcF interval of more than 60 ms compared to their baseline. Forty-one of these also had a prolonged QTcF on day 3 pre-dose 3. There was a significant difference in the mean change of QTcF prolongation between males and females (8. 3 ms, 95% CI: 1. 1, 15. 6). 84% (75/89) were under 12 years old and the maximum age was 37 years. The mean QTcF reduced to 31 ms in both sexes on day 7.

On day 7, seven patients (0.7%) had aQTc interval more than 60 ms above their baseline. Five patients did not have prolongation on day 3 (pre and post dose). Two of the seven patients on day 7, (0.2%) persistently had prolongation of more than 60 ms from day 3 pre- and post- dose. None of the two patients had QTcF readings of more than 500 ms.

## Discussion

This study used CEM, with a very high follow-up rate (>95%) by either home visits or by telephone. Eurartesim® was well tolerated and there was a relatively low rate of AE, 7.3%, of mild or moderate severity. The rate of reported AE in this study was lower than those reported in the previous phase III trials, conducted both in African and Asian populations [14,15,30]. ACT is widely being deployed in endemic countries and used both under prescription and over the counter, and they appear to have a good tolerability. It is not surprising that using a study design simulating the real condition of use, a lower incidence of AE has been reported than in Phase II-III clinical trials, in which usually the anxiety of the patient taking a new drug and the blinded condition in which such drugs are administered may cause an increase of AE reporting. This study demonstrates that a large cohort of patients with confirmed uncomplicated malaria had minimal safety concerns with most events reported being related more to malaria than to intake of Eurartesim®. This provides a significant assurance of the tolerability of Eurartesim® as assessed in real-life conditions in four African countries with patients taking not just the anti-malarial but also other concomitant medications as well.

PQP belongs to the class of bisquinolones, which has been documented to prolong electrocardiographic QT interval and may have significant clinical effect in susceptible individuals. The aim of this study was also to include a nested population of about 1,000 patients, selected according to the ECG criteria, used in previous clinical trials, in which this interval was evaluated in healthy volunteers, to compare the results obtained in this patient population with those obtained in healthy volunteers when a full course of Eurartesim® was administered. The only difference between the nested population and the general population was the exclusion of patients in which baseline QTcF interval was found to be higher than 450 ms. This exclusion criterion was adopted in the selection of the nested population to maintain enrolment criteria similar to those applied to the healthy volunteers in previous studies, in order to be sure to have comparable ECG inclusion criteria. This criterion excluded only 11 out of 1305 patients recruited; therefore this criterion did not really have any impact on selecting a different population in the nested group respect to the general population. The results confirmed that transient prolonged QTcF after intake of Eurartesim® was more common in children less than 12 years and peaked on day 3 when the maximal drug concentration is expected, returning to almost baseline values within four days (day 7 visit). However this period also coincides with the acute period of malaria where evidence of prolongation has been documented [16]. The analysis of the relationship of the time of drug administration with the food intake and concomitant medication will be published after pharmacokinetic analysis. Two cases recorded with increased QTcF > 500 ms on only day 7 may be due to increased heart rate and overestimation of the QT interval or to the influence of the circadian rhythm on QT interval correlated with the different times of ECG collection on day 1,3 and day 7. There was no documentation of clinically relevant cardiac toxicity and no episodes of torsade de pointes or ventricular fibrillation or flutter. This adds to the evidence that a single course of Eurartesim® is safe at therapeutic doses for treating uncomplicated malaria. However, safety data on repeated treatment courses is limited and there might be a potential risk of piperaquine accumulation [31]. Analysis on the biochemical parameters and plasma level concentration of the drug to relate to the QT findings and AE will be published in a subsequent paper. In addition, detailed analysis of the safety data including causality assessment of AE will be undertaken.

This study represents, by far, the largest and most rigorously conducted phase IV assessment of any anti-malarial medicine in real-life conditions in Africa. Previous studies have been limited in terms of numbers [5,6]. This phase IV study of Eurartesim®, carried out relatively quickly after licensure of the drug by the EMA, as part of the risk management plan for Eurartesim®, demonstrates the possibility of undertaking rigorous safety assessment of anti-malarials in real-life settings in malaria-endemic countries. It has produced data that will be contributed to the WHO Programme for International Drug Monitoring and provides useful information for national malaria control programmes that are considering including Eurartesim® as part of their first line treatment of uncomplicated malaria. Together with data from INESS collected since 2009 and on amodiaquine + artesunate (Ghana) and artemether + lumefantrine (Tanzania), this study highlights the usefulness of the INESS platform as an African-led, rigorous scientific platform, working with global partners, to assess the safety and effectiveness of anti-malarial medicines and to provide information on the data collected in a timely manner to all partners.

### Challenges and limitations

There was a delay in initiating the studies due to the long time interval between the submission of the dossier for registration and approval from the various national regulatory authorities. Missed visits or incomplete data created some gaps in collecting information but this was low (<5%) and did not affect the results overall. This was an observational study with no blinding of investigators and participants, therefore, there may have been a tendency to report adverse effects for which symptoms presented at the health facility before treatment as AE which were not “new or worsening events” post-treatment. Another limitation of the study is the incapability to closely estimate the exact QTcF prolongation time shown by some patients in the nested group, with no ECG recordings between days 3 and 7. The repeated blood draw and long waiting time by study participants to complete study procedures in the nested cohort was difficult for sites which were semi-urban, but this was balanced by provision of snacks and meals three to four hours after administration of the drug, ECG procedure and collection ofPKa samples. Re-imbursement of transportation cost and free medical care during the 28 days of participation in the study were also an incentive.

## Conclusion

The study demonstrated that it is feasible to conduct post-licensure safety monitoring of more than 10,000 confirmed malaria patients, including ECG monitoring, of a newly registered anti-malarial, using CEM, as part of pharmacovigilance activities in both rural and semi-urban setting in Africa. The study showed Eurartesim® to be a well-tolerated anti-malarial medicine from the initial analysis though transient QTc prolongation may occur in children under 12 years of age, comparable findings in phase III studies carried out in Africa [14]. This will be analysed further in subsequent papers with availability of pharmacokinetic data and relationship of food intake with the drug. The INESS platform is rigorous and functional and can be used for future drugs and vaccines safety assessment for phase IV studies in Africa.

## Additional files

Additional file 1:
**Definition for safety evaluations.**
Additional file 2:
**List of concomitant medication taken in addition to Eurartesim®, N = 10,591.**
Additional file 3:
**Incidence rate of events reported by SOC preferred term (grouped by MedDRA® coding) in the total cohort (N = 10,925).**
Additional file 4:
**Rates of adverse events by site per 1,000 participants.**
