# Supplementary material for: Prospective observational study to evaluate the clinical safety of the fixed-dose artemisinin-based combination Eurartesim® (dihydroartemisinin/piperaquine), in public health facilities in Burkina Faso, Mozambique, Ghana, and Tanzania
Source: Malar J. 2015 Apr 15;14:160. doi: 10.1186/s12936-015-0664-9 (PMC4405867; doi:10.1186/s12936-015-0664-9)
Supplement: Additional file 1: — Definition for safety evaluations. [file 12936_2015_664_MOESM1_ESM.docx]

Additional file 1

***Definition for safety evaluations:***

*This study used the WHO definition of Adverse Event and Serious Adverse Event*

***Adverse Event (AE)*** *is a sign, symptom, syndrome, disease or biological anomaly suffered by the patient participating in the clinical study and receiving a medicinal product. This term did not imply a causal relationship with the concerned treatment.*

***Serious Adverse Event (SAE)*** *is an adverse event which is life-threatening or necessitates hospitalization or prolongs hospitalization or results in invalidity or long-term or significant disability or is a congenital defect or malformation or is another medically important event or causes death.*
