# Supplementary material for: Prospective observational study to evaluate the clinical safety of the fixed-dose artemisinin-based combination Eurartesim® (dihydroartemisinin/piperaquine), in public health facilities in Burkina Faso, Mozambique, Ghana, and Tanzania
Source: Malar J. 2015 Apr 15;14:160. doi: 10.1186/s12936-015-0664-9 (PMC4405867; doi:10.1186/s12936-015-0664-9)
Supplement: Additional file 2: — List of concomitant medication taken in addition to Eurartesim®, N = 10,591. [file 12936_2015_664_MOESM2_ESM.docx]

Additional file 2

**List of concomitant medication taken in addition to Eurartesim®, N=10,591**

| Concomittant Medication | Frequency | Percent |
| --- | --- | --- |
| Analgesic | 6285 | 56.08 |
| Heamatinic | 1720 | 15.35 |
| Antibiotics | 1451 | 12.95 |
| Expectorant | 480 | 4.28 |
| Antifugal | 329 | 2.94 |
| Oral rehydration salt | 299 | 2.67 |
| Antihelminthic | 261 | 2.33 |
| Antihistamine | 161 | 1.44 |
| Antacid | 91 | 0.81 |
| Intravenous fluid | 31 | 0.28 |
| Antihypertensive | 22 | 0.20 |
| Topical Antibiotic (eye) | 19 | 0.17 |
| Antiemetics | 15 | 0.13 |
| Bronchodialitor | 14 | 0.12 |
| Anti-malarial | 10 | 0.09 |
| H2 Receptor Blocker | 8 | 0.07 |
| Steriod (Skin) | 3 | 0.03 |
| Antipruritic (topical) | 2 | 0.02 |
| Toxoid | 2 | 0.02 |
| Sulphonamide | 1 | 0.01 |
| Anticonvulsant | 1 | 0.01 |
| Antidiabetic | 1 | 0.01 |
| Diuretic | 1 | 0.01 |
| Total | 11207 | 100.00 |
