# Supplementary material for: Prospective observational study to evaluate the clinical safety of the fixed-dose artemisinin-based combination Eurartesim® (dihydroartemisinin/piperaquine), in public health facilities in Burkina Faso, Mozambique, Ghana, and Tanzania
Source: Malar J. 2015 Apr 15;14:160. doi: 10.1186/s12936-015-0664-9 (PMC4405867; doi:10.1186/s12936-015-0664-9)
Supplement: Additional file 3: — Incidence rate of events reported by SOC preferred term (grouped by MedDRA® coding) in the total cohort (N = 10,925). [file 12936_2015_664_MOESM3_ESM.docx]

Additional file 3

Incidence rate of events reported by SOC preferred term (grouped by MedDRA® coding) in the total cohort (N=10,925)

|  |  |  |
| --- | --- | --- |
|  |  |  |
| **MedDRA® System Organ Classification (Preferred Term)**  **N=10, 925** | **Number of events (n)** | **Incidence rate % (n/N)** |
| **Blood and lymphatic system disorders** | **15** | **0.14** |
| **Cardiac disorders** | **5** | **0.05** |
| Palpitations | 4 | 0.04 |
| Other | 1 | 0.01 |
| **Congenital, familial and genetic disorders** | **3** | **0.03** |
| Phimosis | 1 | 0.01 |
| Sickle cell anaemia with crisis | 2 | 0.02 |
| **Ear and labyrinth disorders** | **2** | **0.02** |
| Ear pain | 1 | 0.01 |
| Othrrhoea | 1 | 0.01 |
| **Eye disorders** | **8** | **0.07** |
| Conjunctivitis | 7 | 0.06 |
| Photophobia | 1 | 0.01 |
| **Gastrointestinal disorders** | **150** | **1.37** |
| Abdominal distension | 1 | 0.01 |
| Abdominal pain | 17 | 0.16 |
| Abdominal pain upper | 2 | 0.02 |
| Diarrhoea | 29 | 0.27 |
| Enteritis | 4 | 0.04 |
| Food poisoning | 1 | 0.01 |
| Gastric ulcer | 1 | 0.01 |
| Gastritis | 9 | 0.08 |
| Gastro duodenal ulcer | 1 | 0.01 |
| Gingivitis | 1 | 0.01 |
| Glossodynia | 1 | 0.01 |
| Nausea | 9 | 0.08 |
| Pain | 1 | 0.01 |
| Stomatitis | 5 | 0.05 |
| Vomiting | 68 | 0.62 |
| **General disorders and administrative site conditions** | **83** | **0.76** |
| Asthenia | 13 | 0.12 |
| Chest discomfort | 1 | 0.01 |
| Chest pain | 3 | 0.03 |
| Chills | 1 | 0.01 |
| Discomfort | 1 | 0.01 |
| Face oedema | 2 | 0.02 |
| Fatigue | 2 | 0.02 |
| Malaise | 1 | 0.01 |
| Pain | 6 | 0.05 |
| Pyrexia | 53 | 0.49 |
| **Immune system disorders** | **1** | **0.01** |
| Eye allergy | 1 | 0.01 |
| **Infections and infestations** | **354** | **3.24** |
| Abscess | 1 | 0.01 |
| Amoebic dysentery | 5 | 0.05 |
| Arthritis | 1 | 0.01 |
| Body tinea | 1 | 0.01 |
| Bronchiolitis | 1 | 0.01 |
| Bronchitis | 15 | 0.14 |
| Bullous impetigo | 1 | 0.01 |
| Cellulitis | 2 | 0.02 |
| Ear infection | 1 | 0.01 |
| Fungal infection | 1 | 0.01 |
| Fungal skin infection | 3 | 0.03 |
| Gastroenteritis | 41 | 0.38 |
| Helminthic infection | 16 | 0.15 |
| Hepatitis | 1 | 0.01 |
| Herpes virus infection | 1 | 0.01 |
| Impetigo | 2 | 0.02 |
| Infections and infestations | 1 | 0.01 |
| Influenza | 3 | 0.03 |
| Malaria | 84 | 0.77 |
| Mastitis | 1 | 0.01 |
| Meningitis | 1 | 0.01 |
| Meningitis bacterial | 1 | 0.01 |
| Meningitis meningococcal | 1 | 0.01 |
| Mumps | 1 | 0.01 |
| Oral herpes | 1 | 0.01 |
| Orchitis | 1 | 0.01 |
| Osteoarthritis | 1 | 0.01 |
| Otitis media | 5 | 0.05 |
| Pelvic inflammatory disease | 1 | 0.01 |
| Pharyngitis | 1 | 0.01 |
| Plasmodia infections | 4 | 0.04 |
| Pneumonia | 23 | 0.21 |
| Respiratory tract infection | 1 | 0.01 |
| Rhinitis | 21 | 0.19 |
| Sepsis | 4 | 0.04 |
| Subcutaneous abscess | 1 | 0.01 |
| Tinea capitis | 1 | 0.01 |
| Tinea infection | 1 | 0.01 |
| Tonsillitis | 1 | 0.01 |
| Tooth abscess | 1 | 0.01 |
| Tooth infection | 1 | 0.01 |
| Trichomoniasis | 1 | 0.01 |
| Tuberculosis | 1 | 0.01 |
| Typhoid fever | 1 | 0.01 |
| Upper respiratory tract infection | 80 | 0.73 |
| Urinary tract infection | 12 | 0.11 |
| Vulvovaginal candidiasis | 3 | 0.03 |
| **Injury, poisoning and procedural complications** | **5** | **0.05** |
| Contusion | 1 | 0.01 |
| Non-site specific injuries | 2 | 0.02 |
| Wound | 2 | 0.02 |
| **Metabolism and nutrition disorders** | **14** | **0.13** |
| Decreased appetite | 13 | 0.12 |
| Hypoglycaemia | 1 | 0.01 |
| **Musculoskeletal and connective tissue disorders** | **12** | **0.11** |
| Arthralgia | 1 | 0.01 |
| Arthritis | 1 | 0.01 |
| Back pain | 4 | 0.04 |
| Musculoskeletal pain | 5 | 0.05 |
| Myalgia | 1 | 0.01 |
| **Nervous system disorders** | **50** | **0.46** |
| Convulsion | 4 | 0.04 |
| Dizziness | 8 | 0.07 |
| Headache | 31 | 0.28 |
| Neuropathy peripheral | 1 | 0.01 |
| Sedation | 1 | 0.01 |
| Somnolence | 4 | 0.04 |
| Tremor | 1 | 0.01 |
| **Pregnancy, puerperium and perinal conditions** | **1** | **0.01** |
| Pregnancy | 1 | 0.01 |
| **Psychiatric disorders** | **4** | **0.04** |
| Anxiety disorder | 1 | 0.01 |
| Insomnia | 1 | 0.01 |
| Restlessness | 1 | 0.01 |
| **Renal and urinary disorders** | **2** | **0.02** |
| Glomerulonephritis acute | 1 | 0.01 |
| Renal failure acute | 1 | 0.01 |
| **Reproductive system and breast disorders** | **2** | **0.02** |
| Intrauterine death | 1 | 0.01 |
| Menorrhagia | 1 | 0.01 |
| **Respiratory, thoracic and mediastinal disorders** | **59** | **0.54** |
| Asthma | 2 | 0.02 |
| Cough | 53 | 0.49 |
| Dyspnoea | 1 | 0.01 |
| Epistaxis | 1 | 0.01 |
| Hiccups | 1 | 0.01 |
| Pleurisy | 1 | 0.01 |
| **Skin and subcutaneous tissue disorders** | **26** | **0.24** |
| Dermatitis | 2 | 0.02 |
| Dermatosis | 1 | 0.01 |
| Pruritus | 13 | 0.12 |
| Rash | 6 | 0.05 |
| Skin lesion | 1 | 0.01 |
| Swelling face | 2 | 0.02 |
| Urticaria | 1 | 0.01 |
| **Other** | **1** | **0.01** |
| Sexual assault | 1 | 0.01 |
