# Supplementary material for: Prospective observational study to evaluate the clinical safety of the fixed-dose artemisinin-based combination Eurartesim® (dihydroartemisinin/piperaquine), in public health facilities in Burkina Faso, Mozambique, Ghana, and Tanzania
Source: Malar J. 2015 Apr 15;14:160. doi: 10.1186/s12936-015-0664-9 (PMC4405867; doi:10.1186/s12936-015-0664-9)
Supplement: Additional file 4: — Rates of adverse events by site per 1,000 participants. [file 12936_2015_664_MOESM4_ESM.docx]

Additional file 4:

Rates of adverse events by site per 1,000 participants

| **MedDRA® System Organ Classification** | **Study Sites** | | | | | | | **Total**  **N=10925** |
| --- | --- | --- | --- | --- | --- | --- | --- | --- |
|  | **Dodowa**  **n=899** | **Kintampo**  **n=1875** | **Navrongo**  **n=1911** | **Nouna**  **n=1803** | **Rufiji**  **n=1098** | **Manhica**  **n=2062** | **Nanoro**  **n=1277** |  |
| Blood and lymphatic system disorders | 1 (1.1) | 1 (0.5) | 2 (1.0) | 0 (0.00) | 1 (0.9) | 8 (3.9) | 2 (16) | 15 (1.4) |
| Cardiac disorders | 0 (0.0) | 2 (1.1) | 0 (0.0) | 0 (0.0) | 1 (0.9) | 2 (1.0) | 0 (0.0) | 5 (0.5) |
| Congenital, familial and genetic disorders | 0 (0.0) | 2 (1.1) | 0 (0.0) | 0 (0.0) | 0 (0.0) | 0 (0.0) | 1 (0.8) | 3 (0.3) |
| Ear and labyrinth disorders | 0 (0.0) | 0 (0.0) | 0 (0.0) | 2 (1.1) | 0 (0.0) | 0 (0.0) | 0 (0.0) | 2 (0.2) |
| Eye disorders | 0 (0.0) | 1 (0.5) | 2 (1.0) | 2 (1.1) | 1 (0.9) | 1 (0.5) | 1 (0.8) | 8 (0.7) |
| Gastrointestinal disorders | 24 (26.7) | 13 (6.9) | 10 (5.2) | 55 (30.5) | 40 (36.4) | 3 (1.5) | 5 (3.9) | 150 (13.7) |
| General disorders and administrative site conditions | 8 (8.9) | 3 (1.6) | 10 (5.2) | 9 (5.0) | 23 (20.9) | 26 (12.6) | 4 (3.1) | 83 (7.6) |
| Immune system disorders | 0 (0.0) | 0 (0.0) | 1 (0.5) | 0 (0.0) | 0 (0.0) | 0 (0.0) | 0 (0.0) | 1 (0.1) |
| Infections and infestations | 27 (30.0) | 61 (32.5) | 123 (64.4) | 26 (14.4) | 41 (37.3) | 17 (8.2) | 59 (46.2) | 354 (32.4) |
| Injury, poisoning and procedural complications | 0 (0.0) | 0 (0.0) | 2 (1.0) | 2 (1.1) | 1 (0.9) | 0 (0.0) | 0 (0.0) | 5 (0.5) |
| Metabolism and nutrition disorders | 0 (0.0) | 0 (0.0) | 2 (1.0) | 1 (0.6) | 7 (6.4) | 4 (1.9) | 0 (0.0) | 14 (1.3) |
| Musculoskeletal and connective tissue disorders | 4 (4.4) | 0 (0.0) | 5 (2.6) | 1 (0.6) | 0 (0.0) | 1 (0.5) | 1 (0.8) | 12 (1.1) |
| Nervous system disorders | 6 (6.7) | 4 (2.1) | 3 (1.6) | 17 (9.4) | 12 (10.9) | 8 (3.9) | 0 (0.0) | 50 (4.6) |
| Pregnancy, puerperium and perinal conditions | 0 (0.0) | 0 (0.0) | 1 (0.5) | 0 (0.0) | 0 (0.0) | 0 (0.0) | 0 (0.0) | 1 (0.1) |
| Psychiatric disorders | 0 (0.0) | 0 (0.0) | 2 (0.1) | 1 (0.6) | 1 (0.9) | 0 (0.0) | 0 (0.0) | 4 (0.4) |
| Renal and urinary disorders | 0 (0.0) | 1 (0.5) | 1 (0.1) | 0 (0.0) | 0 (0.0) | 0 (0.0) | 0 (0.0) | 2 (0.2) |
| Reproductive system and breast disorders | 0 (0.0) | 1 (0.5) | 0 (0.0) | 0 (0.0) | 0 (0.0) | 0 (0.0) | 1 (0.8) | 2 (0.2) |
| Respiratory, thoracic and mediastinal disorders | 1 (1.1) | 4 (2.1) | 5 (2.6) | 11 (6.1) | 12 (10.9) | 24 (11.6) | 2 (1.6) | 59 (5.4) |
| Skin and subcutaneous tissue disorders | 4 (4.4) | 3 (1.6) | 6 (3.1) | 3 (1.7) | 7 (6.4) | 1 (0.5) | 2 (1.6) | 26 (2.4) |
| Other | 0 (0.0) | 0 (0.0) | 0 (0.0) | 0 (0.0) | 1 (0.9) | 0 (0.0) | 0 (0.0) | 1 (0.1) |
| **Total** | **75** (83.4) | **96** (51.2) | **175** (91.6) | **130** (72.1) | 148(134.8) | **95** (46.1) | **78** (61.1) | **797** (73.0) |
